# Expanding molecular and clinical spectrum of CPT1C‐associated hereditary spastic paraplegia (SPG73)—a case series

**DOI:** 10.1002/acn3.52288

**Published:** 2024-12-29

**Authors:** Alexandra K. Brooks, Vicente Quiroz, Luca Schierbaum, Amy Tam, Julian E. Alecu, Darius Ebrahimi‐Fakhari

**Affiliations:** ^1^ Department of Neurology, Movement Disorders Program Boston Children's Hospital, Harvard Medical School Boston Massachusetts USA; ^2^ Department of General Pediatrics Boston Children's Hospital, Harvard Medical School Boston Massachusetts USA

## Abstract

Autosomal‐dominant variants in the *CPT1C* gene have been associated with hereditary spastic paraplegia type 73 (SPG73), which typically presents with slowly progressive lower limb weakness and spasticity and is therefore considered a pure form of hereditary spastic paraplegia. However, we report two unrelated males with novel *CPT1C* variants (NM_001199753.2: patient 1: c.2057_2061del (p.Ile686SerfsTer8) and patient 2: c.2020‐1G>C (p.?)) who presented with lower limb spasticity at 4 and 3 years old, respectively. Both patients also experienced significant cognitive impairment, seizures, or neurobehavioral symptoms. These cases illustrate a broader and more complex clinical spectrum of SPG73, extending beyond the traditionally recognized pure motor symptoms.

## Introduction

The hereditary spastic paraplegias (HSPs) are an expanding group of monogenic disorders characterized by progressive spasticity and weakness of the lower limbs.^1^ HSPs are traditionally categorized as either pure or complicated HSP based on the presence or absence of additional neurological manifestations, including cognitive dysfunction, peripheral neuropathy, or bulbar impairment.^1^, ^2^

Individuals presenting with symptoms of HSP in early childhood continue to be misdiagnosed with cerebral palsy due to overlapping clinical features and in many cases, slow initial disease progression.^1^ Advancements in the accessibility of genetic testing over the past decade have facilitated accurate diagnosis and differentiation between these conditions.^1^, ^2^

To date, there are over 80 different subtypes of HSP, with an increasing number of associated genetic loci. The most prevalent subtype is pure adult‐onset autosomal dominant HSP, which stems from variants in various genes including *SPAST* (SPG4), *ATL1* (SPG3A), and *REEP1* (SPG31).

Over the past decade, a total of three studies have identified variants in the carnitine palmitoyl‐transferase (*CPT1C*) gene as the cause of hereditary spastic paraplegia type 73 (SPG73),^3^ categorized as a pure autosomal dominant form.^2^, ^4^, ^5^ The first previously identified variant was found to impair regulatory/catalytic protein domains, indicating compromised CPT1C function.^4^ In contrast, other variants led to nonsense‐mediated mRNA decay, with neither full‐length nor truncated proteins detected.^2^, ^5^ The predominant clinical manifestations have been slowly progressive lower limb weakness and spasticity, indicative of a gradually evolving clinical course.^4^, ^5^ Reported patients exhibited normal cognitive function with no documented behavioral concerns, suggesting that *CPT1C* variants typically result in an adult‐onset pure motor form of HSP.^4^, ^5^ Nevertheless, more recent reports describe two additional patients who presented with a complex form of HSP, accompanied by seizures or visual impairment.^2^ Notably, similar to previous cases, these patients also exhibited normal cognitive functioning and no behavioral issues.^2^ Despite only a few reported cases, these findings underscore the expanding genetic complexity of HSP and the need for further research into its underlying mechanisms.

In this study, we present two families with novel variants in *CPT1C*. Our patients are both male individuals who inherited a Pathogenic (Patient 1) or Likely Pathogenic (Patient 2) variant in *CPT1C* from their mothers, who also displayed lower leg cramps and gait impairment. Both patients presented with early‐onset and progressive lower limb spasticity with mild–to‐moderate impairment of gait. Remarkably, our patients also exhibited cognitive impairments and significant neurobehavioral concerns, which have not been described prior.

## Case 1

The patient is a 10‐year‐old male with an unremarkable prenatal history. He reached appropriate developmental milestones of sitting unsupported at 6 months, standing unsupported at 9 months, and walking unsupported at 11 months. At the age of 2, his parents noticed his legs crossing. By ages 4–5, the family observed increased clumsiness and frequent falls, attributed to dragging or tripping over his feet. While he was able to run and play adequately, the family noted morning leg tightness and difficulties with fine motor skills. His initial evaluation revealed bilateral lower leg spasticity, ankle clonus, a positive Babinski sign, and an abnormal, unsteady gait (GMFCS 1). Notably, there was no muscle wasting, urinary or stool incontinence, or neuropathy. MR imaging of the brain showed a Chiari 1 malformation but was otherwise unremarkable.

**Figure 1:**
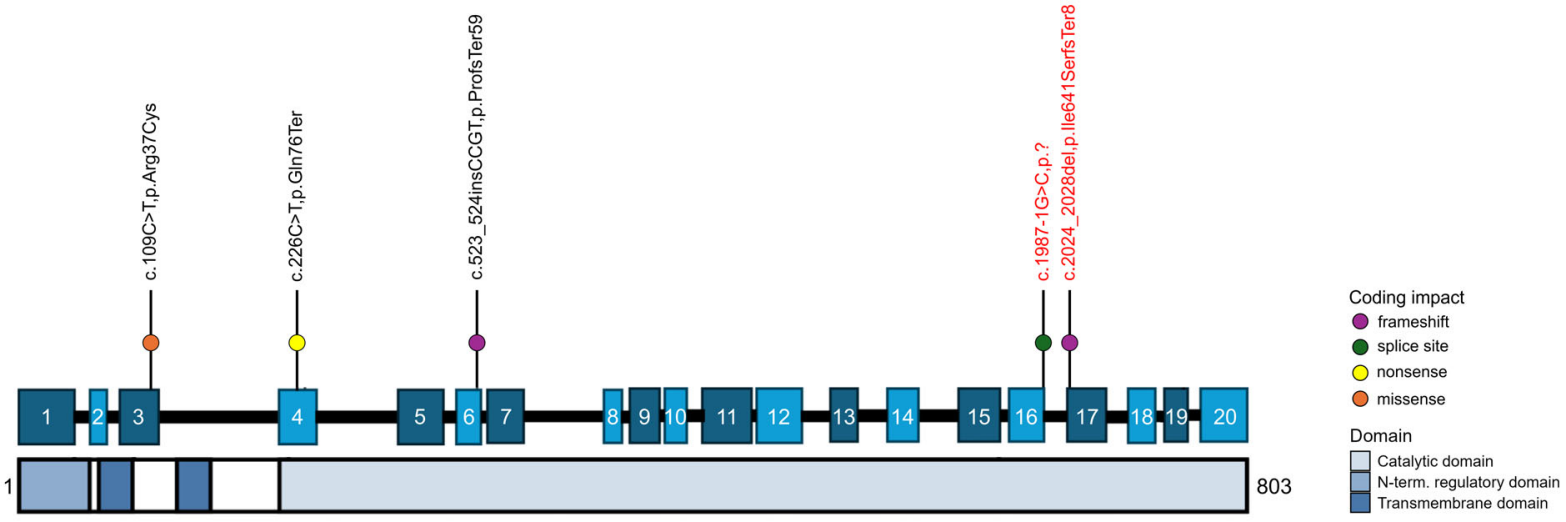
*CPT1C* (NM_001199753.2) variant distribution. Schematic of documented variant locations on gene and protein structure with colored dots representing coding impact. Novel variants are labeled in red.

Variant c.2057_2061del (Ile686SerfsTer8) in *CPT1C* (NM_001199753.2) was identified through multi‐gene panel testing and classified as Pathogenic (Fig. 1). This variant was found to be inherited from his mother who had also been experiencing bilateral leg cramps and impaired vibration sensation in both feet over the past year. There was no additional developmental history available for the mother, and the rest of the family history was otherwise unremarkable.

Approximately 2 years after the molecular diagnosis, the family noted worsening spasticity. On examination, his Spastic Paraplegia Rating Scale (SPRS) score was 8, indicating mild gait impairment and lower extremity muscle weakness and spasticity (Table 1). Spasticity is currently managed with baclofen and physical therapy.

**Table 1:** Comprehensive summary of genotypic and phenotypic data for all published and current CPT1C/SPG73 cases.

Patient	Family 1 (Rinaldi et al. 2016)	Family 2 (Hong et al. 2019)	Case 3 (Wang et al 2022)	Case 4 (Wang et al. 2022)	Case 5	Case 6
Variant (NM_001199753.2)	c.109C>T, p.Arg37Cys	c.226C>T, p.Gln76Ter	c.524_527dup, p. Pro177ArgfsTer59	c.226C>T, p.Gln76Ter	c.2057_2061del, p.Ile686SerfsTer8	c.2020‐1G>C, p.?
Inheritance	AD	AD—maternal	AD—maternal	AD—paternal	AD—maternal	AD—maternal
Affected family	Multi‐generational family with 6 affected individuals	Mother asymptomatic	Mother asymptomatic	Father asymptomatic	Mother symptomatic with worsening bilateral leg cramps and impaired vibration sensation	Mother symptomatic from age 14, with progressively worsening cramping, pain, spasticity in lower extremities, leading to frequent falls
Sex	3M, 3F	2F, 1M	M	M	M	M
Age of presentation	Adult onset (19–48 years)	Infancy for both children	Infancy (age 2)	Infancy (from birth)	Infancy (age 4)	Infancy (age 3)
Progression	Slowly progressive—ranging from mild impairment to loss of ambulation 10–15 years after initial symptoms	Both children with reversible progression by age 10	Independently walk with abnormal gait	1 year old at follow up—cannot walk or hold head up currently	Progressive spasticity but walking without aid	Progressive weakness and spasticity requiring wheelchair for ambulation
Spasticity	Progressed to moderate/severe	+	+	+	Mild	Moderate
Weakness	Mild	+	−	+	Mild	Moderate
developmental delay	None	Delayed walking milestone (achieved independent walking at age 3 and 3.5 years)	Delay of gross motor and speech	Severely delayed milestones	Reached initial gross motor, then developed spasticity	Gross motor and speech delay
Cognition	Normal	Normal	Normal	Normal	Normal	Moderate intellectual disability with deficits in working memory, processing speed, and notable impairment in adaptive behaviors
MRI brain	Normal	Normal	Cortical dysplasia	Thinning of corpus callosum; abnormal signal of bilateral paraventricular; enlargement of bilateral ventricles	Mild Chiari 1 malformation, but otherwise unremarkable	Normal
Seizures	No	No	+	No	No	+
Behavior	−	−	−	−	ADHD, anxiety disorder	ADHD, anxiety disorder, autism spectrum disorder, depressive disorder, disruptive mood dysregulation disorder, conduct disorder

Notably, the patient has exhibited separation anxiety from a young age, with continued significant anxiety as he aged leading to a formal diagnosis of anxiety disorder. Further evaluation led to a diagnosis of attention‐deficit/hyperactivity disorder. Current medications include amphetamine/dextroamphetamine (Adderall) and sertraline.

## Case 2

The patient is a 17‐year‐old male with an unremarkable prenatal history. He initially met developmental milestones but began experiencing muscle cramps in his legs around age 3, which progressed to lower limb spasticity by age 8. By age 14, progressive spasticity affected both upper and lower extremities, leading to contractures of the ankles, muscle wasting, and significant spontaneous ankle clonus (GMFCS 2–3).

Genetic testing identified a likely pathogenic variant in *CPT1C* (NM_001199753.2: c.2020‐1G>C) (Fig. 1), inherited from his mother, who self‐reportedly met all neurodevelopmental milestones as a child but then started to exhibit neurological symptoms beginning around age 14–15. These included progressive spasticity, bilateral lower extremity muscle cramps, nocturnal pain, and frequent falls. His maternal grandfather also had comparable walking difficulties (File S1).

On follow‐up at age 18, the patient's SPRS score increased from 16 to 17, indicating moderate walking impairment and lower extremity spasticity. He now requires orthotics and wheelchair assistance for longer distances (GMFCS 3–4) (Table 1).

Interestingly, the patient also has a history of generalized tonic–clonic seizures, first occurring at age 5, with EEG findings revealing sharp waves in the temporal region. Antiseizure medications were initially prescribed but discontinued after 2 years of seizure freedom.

In addition to his neurological challenges, the patient faces significant cognitive difficulties, with a history of aggressive and impulsive behavior from a young age. His IQ testing suggests moderate cognitive impairment (full scale IQ: 57), with notable deficits in working memory and processing speed. An adaptive behavior score of 54 indicates severe difficulties in daily living and socialization. Diagnoses include ADHD, anxiety disorder, depressive disorder, disruptive mood dysregulation disorder, conduct disorder, and autism spectrum disorder.

Current pharmacotherapy includes sertraline, lurasidone, and olanzapine, which appear effective. However, the cognitive and behavioral issues continue to significantly impact his daily life.

## Discussion

In this report, we describe two unrelated male patients with inherited, novel, pathogenic *CPT1C* variants. Consistent with previous observations of *CPT1C* variants, both patients showed progressive lower limb spasticity, with the second individual exhibiting moderate impairment in mobility that required the use of a wheelchair for longer distances. Notably, both patients also presented with a range of neurological symptoms beyond those typically associated with *CPT1C* variants, including cognitive impairment, seizures, neurobehavioral disorders, and psychiatric symptoms. Notably, case 2 displayed significant cognitive deficits, poor adaptive behaviors with significant difficulties in daily living skills, low IQ, and severe behavioral dysregulation. Both patients also had neurodevelopmental comorbidities such as ADHD and ASD, with Patient 2 additionally experiencing concurrent mood disorders including anxiety disorder, depressive disorder, disruptive mood dysregulation disorder, and conduct disorder. These cases highlight a more complex manifestation of HSP than the previously described benign clinical course typically associated with *CPT1C* variants, broadening the understanding of the clinical phenotype linked to SPG73 with its diverse manifestations.

The literature describes four distinct families with documented variants of *CPT1C*, with details listed in Table 1. The first family, detailed by Rinaldi et al. in 2016,^4^ is a multi‐generational family comprising six individuals who experienced adult‐onset lower limb spasticity and weakness, with notably slow disease progression. In contrast, three other families reported symptoms beginning in infancy.^2^, ^5^ Family 2, in particular, exhibited a reversible progression of symptoms, whereas Family 3 experienced only minimal gait impairment. The fourth case involved a patient who faced significant motor delays, unable to hold up their head at 1 year of age; however, there is limited information on subsequent progression. Of the 4 cases, cases 3 and 4 were diagnosed with complex HSP due to seizures and vision impairment, respectively. Overall, all affected individuals in these families exhibited normal cognitive function and no behavioral abnormalities, a significant difference from the two families in our study. In contrast, existing literature on other families with *CPT1C* variants demonstrates mild symptom severity, with many cases exhibiting mild motor issues with normal cognitive function and no neurobehavioral issues, highlighting the distinct clinical profile of our patients.

The two cases presented here feature novel, Pathogenic and Likely Pathogenic *CPT1C* variants located toward the end of the gene, a frameshift deletion and splice‐site mutation, respectively. Of note, the observed phenotypic variability within families, especially among mothers who seem to exhibit mild symptoms, may be influenced by factors such as environmental stressors. Additionally, the presence of other genetic modifiers could contribute to incomplete penetrance and variable expressivity of the clinical phenotype within families. Previous research has linked loss‐of‐function (LoF) variants in *CTP1C* to a decrease in both the number and size of lipid droplets (LD).^4^ This is particularly significant given that corticospinal neurons, where *CPT1C* is expressed in the brain and spinal cord, are especially vulnerable to disruptions in LD metabolism.^3^ Moreover, the pathogenesis of HSP is associated with impairments in LD biogenesis. Our identified LoF variants, which result in notable frameshift and splice site changes, are likely to result in significant protein dysfunction, further reducing LDs. Due to the limitation of available tissue samples, functional analysis of the *CPT1C* variants in Patient 1 and Patient 2 was not possible, however, this remains a key focus for future research. Overall, these cases expand the understanding of the clinical phenotype of SPG73 and patients with *CPT1C* variants, underscoring the need for further investigation into their diverse manifestations.

## Author Contributions

AKB, VQ, and DEF conceptualized the study. AM, VQ, LS, and DEF provided cohort data. JEA and LS created Fig. 1. AKB performed the literature review and drafted the original manuscript. All authors contributed to editing the final draft of the manuscript.

## Conflicts of Interest

The authors declare no conflicts of interest.

## Supporting information


**File S1.** Pedigree of family 2.

## Data Availability

Data are available from the corresponding author upon reasonable request.

## References

[1] Parodi L , Coarelli G , Stevanin G , et al. Hereditary ataxias and paraparesias: clinical and genetic update. Curr Opin Neurol. 2018;31:462‐471. doi:10.1097/WCO.0000000000000585 29847346

[2] Wang J , Fang F , Ding C , et al. Clinical and genetic spectrum of hereditary spastic paraplegia in Chinese children. Dev Med Child Neurol. 2023;65:416‐423. doi:10.1111/dmcn.15385 36109173

[3] Casals N , Zammit V , Herrero L , et al. Carnitine palmitoyltransferase 1C: from cognition to cancer. Prog Lipid Res. 2016;61:134‐148. doi:10.1016/j.plipres.2015.11.004 26708865

[4] Rinaldi C , Schmidt T , Situ AJ , et al. Mutation in CPT1C associated with pure autosomal dominant spastic paraplegia. JAMA Neurol. 2015;72:561‐570. doi:10.1001/jamaneurol.2014.4769 25751282 PMC5612424

[5] Hong D , Cong L , Zhong S , et al. A novel CPT1C variant causes pure hereditary spastic paraplegia with benign clinical course. Ann Clin Transl Neurol. 2019;6:610‐614. doi:10.1002/acn3.717 30911584 PMC6414484

