# Supplementary material for: Expanding molecular and clinical spectrum of CPT1C‐associated hereditary spastic paraplegia (SPG73)—a case series
Source: Ann Clin Transl Neurol. 2024 Dec 29;12(3):648–52. doi: 10.1002/acn3.52288 (PMC11920729; doi:10.1002/acn3.52288)
Supplement: Supplementary file 1 — File S1. Pedigree of family 2. [file ACN3-12-648-s001.pdf]

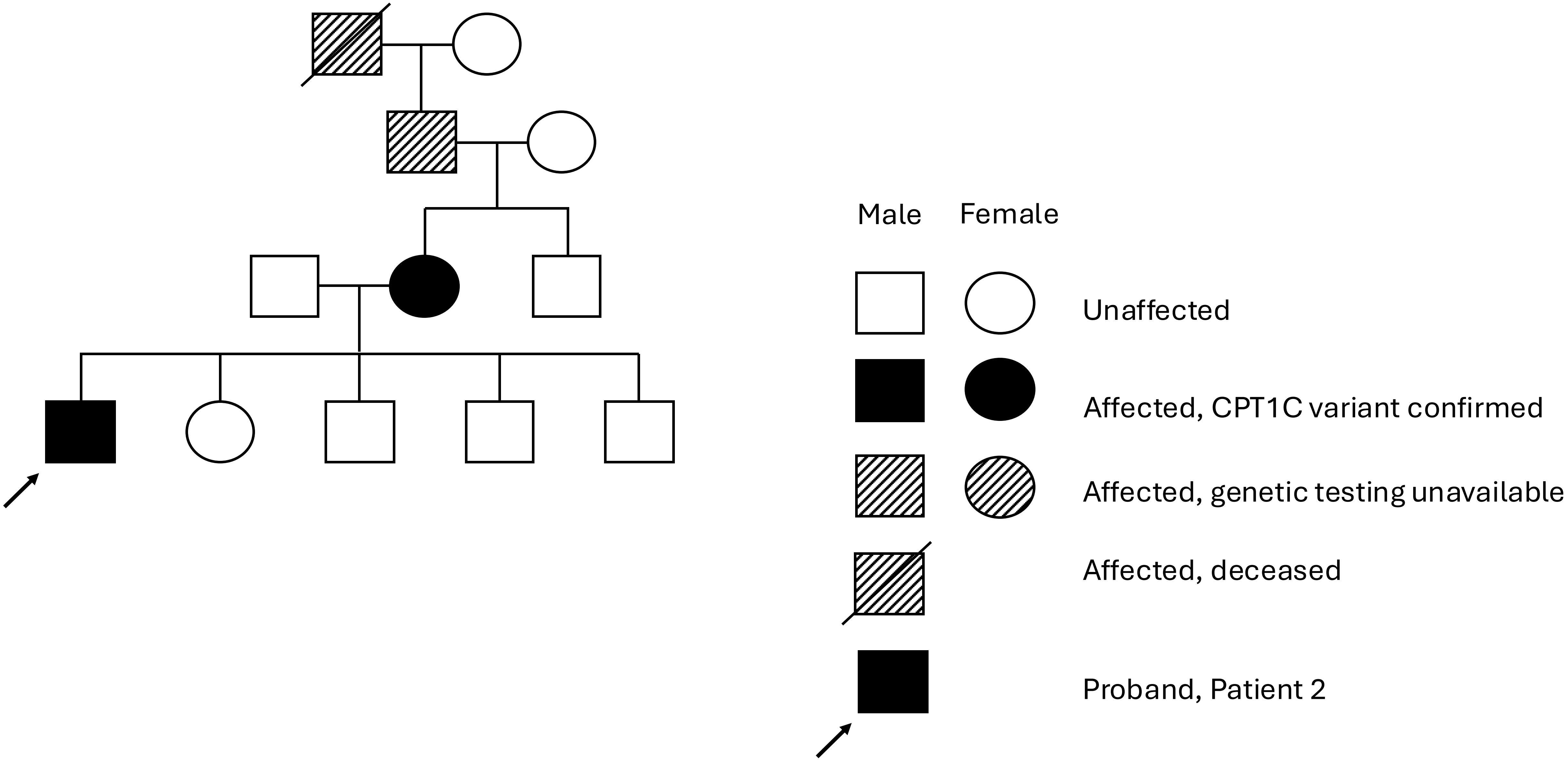
Male
Female
Unaffected
Affected, CPT1C variant confirmed
Affected, genetic testing unavailable
Affected, deceased
Proband, Patient 2
